# Supplementary material for: First Report of the Emerging Pathogen Kodamaea ohmeri in Honduras
Source: J Fungi (Basel). 2024 Feb 28;10(3):186. doi: 10.3390/jof10030186 (PMC10971700; doi:10.3390/jof10030186)
Supplement: Supplementary file 1 [file jof-10-00186-s001.zip › jof-2876939-supplementary.pdf]

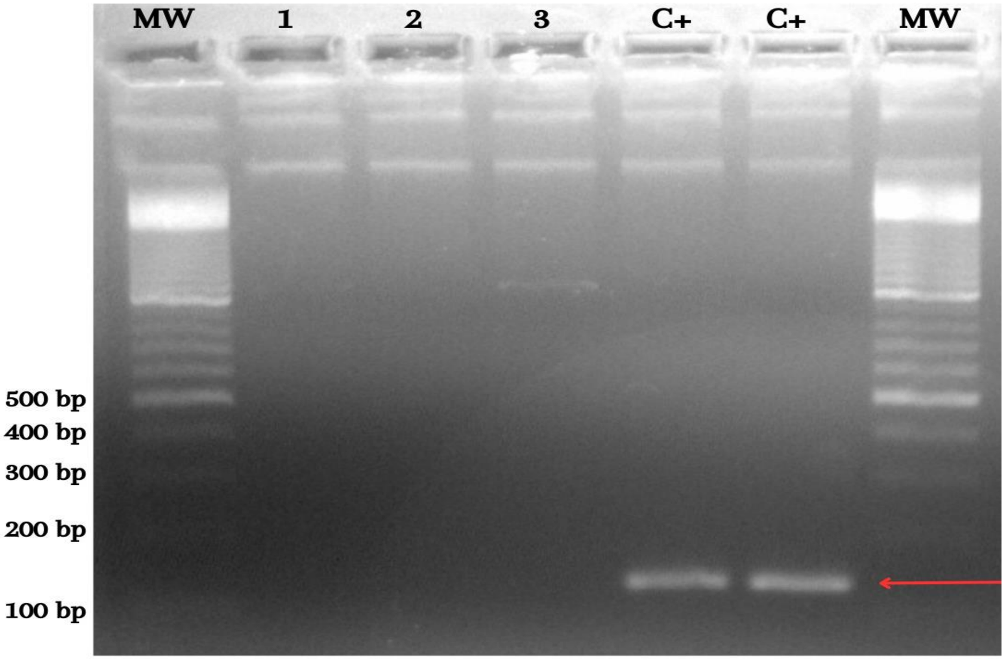

Figure S1. Agarose gel electrophoresis. Lanes named as C+ show a 137 bp band as a result of amplification of the GPI gene from the *C. auris* reference strain. Lanes 1, 2 and 3 isolates of *K. ohmeri*, no amplification products are observed. Molecular weight marker from lanes 1 and 7 (size 100 bp).

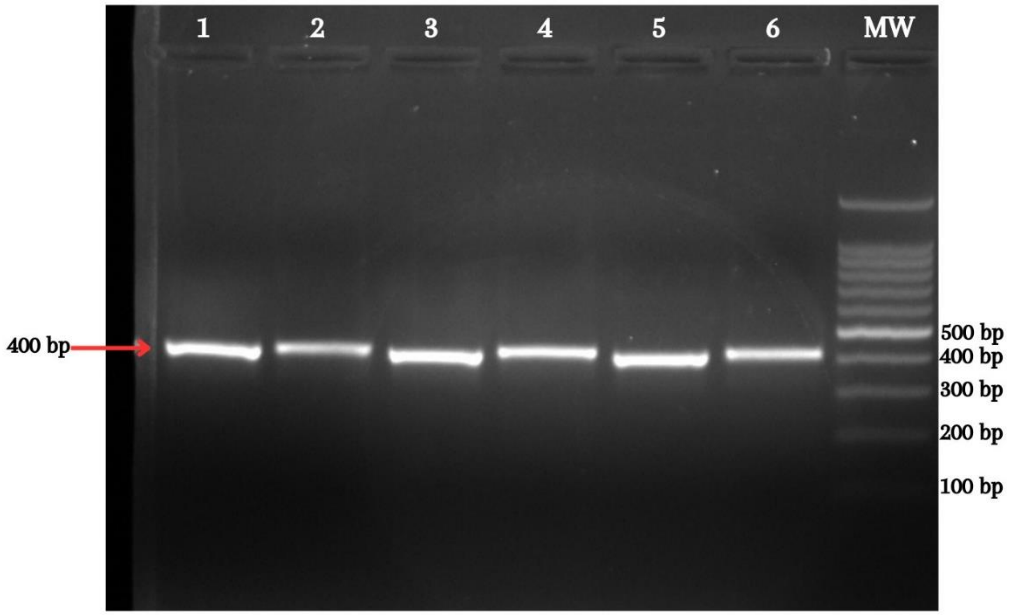

Figure S2. Agarose gel electrophoresis. Lanes 1, 3, and 5 show the amplification products of the internal transcribed spacer (ITS) of the ribosomal region of *K. ohmeri*. Lanes 2, 4, and 6 show the PCR products after being subjected to the MspI enzyme. Molecular weight marker lane 7 (size 100 bp).

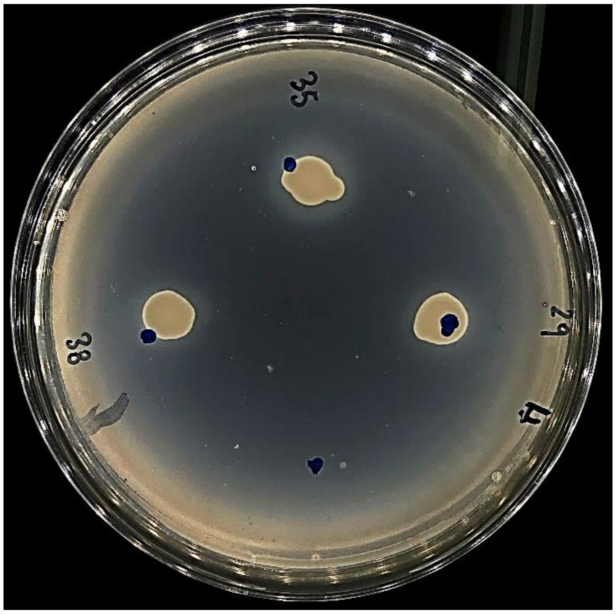

Figure S3. Determination of phospholipase activity of *Kodamaea ohmeri* isolates on Sabouraud agar supplemented with egg yolk.

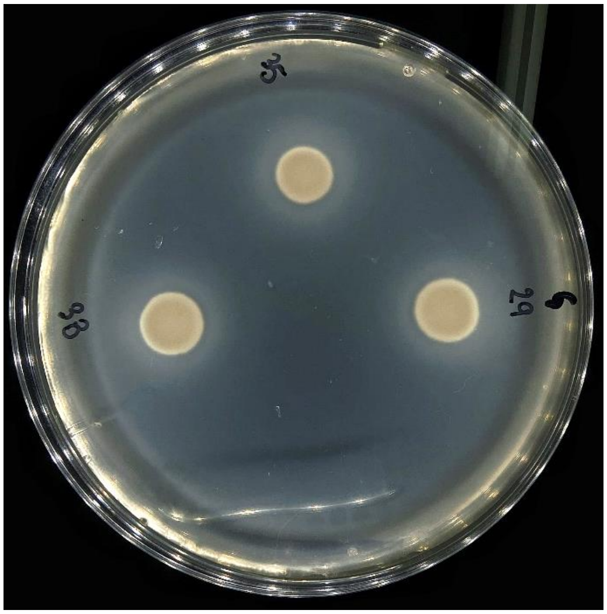

Figure S4. Determination of the protease activity of *Kodamaea ohmeri* isolates, gelatinase test.

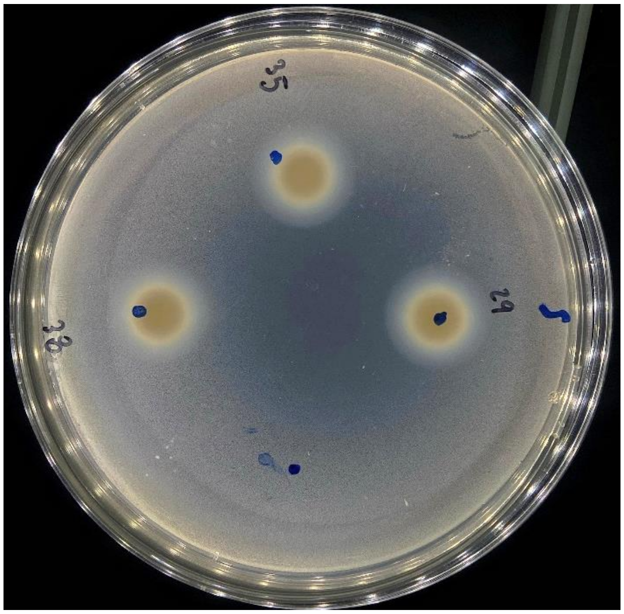

Figure S5. Determination of the protease activity of *Kodamaea ohmeri* isolates, Bovine serum albumin agar (BSA).

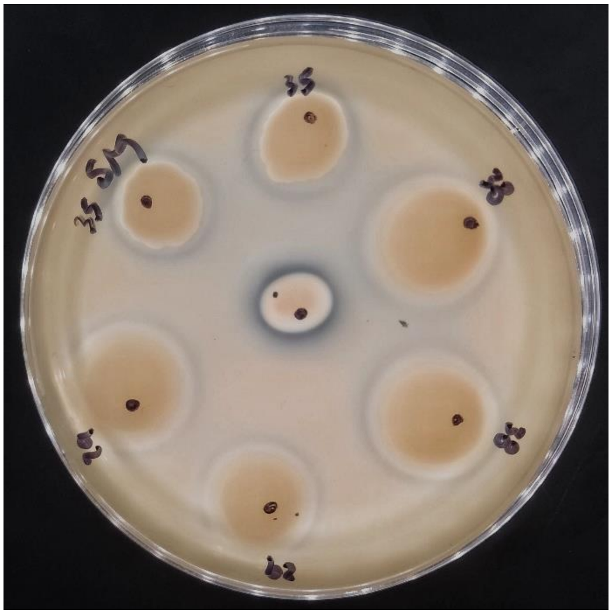

Figure S6. Determination of the protease activity of *Kodamaea ohmeri* isolates. Caseinase activity.

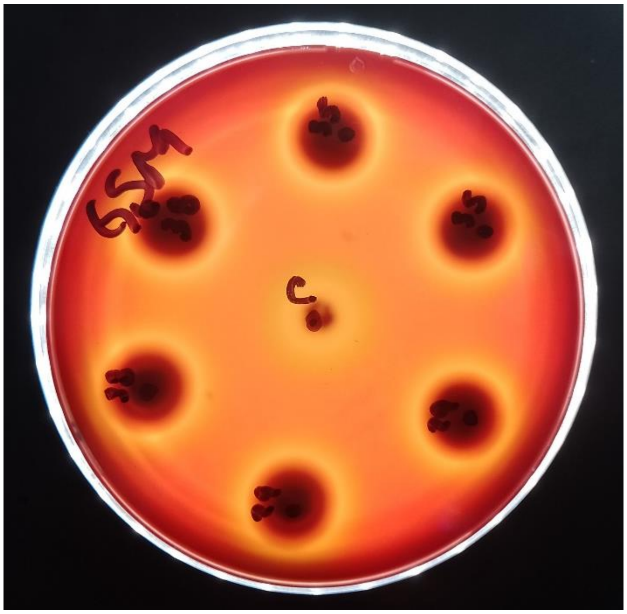

Figure S7. Determination of the hemolytic activity of *Kodamaea ohmeri* isolates. Test on Sabouraud agar supplemented with 6% human blood and 3% glucose (pH = 5.6).
